# SCENERY: a web application for (causal) network reconstruction from cytometry data

**DOI:** 10.1093/nar/gkx448

**Published:** 2017-05-19

**Authors:** Georgios Papoutsoglou, Giorgos Athineou, Vincenzo Lagani, Iordanis Xanthopoulos, Angelika Schmidt, Szabolcs Éliás, Jesper Tegnér, Ioannis Tsamardinos

**Affiliations:** 1 Computer Science Department, University of Crete, Heraklion, Crete 700 13, Greece; 2 Gnosis Data Analysis I.K.E., Heraklion, Crete 71305, Greece; 3 Unit of Computational Medicine, Center for Molecular Medicine, Department of Medicine Solna, Karolinska University Hospital and Science for Life Laboratory, Karolinska Institutet, Stockholm 171 76, Sweden; 4 Biological and Environmental Sciences and Engineering Division, Computer, Electrical and Mathematical Sciences and Engineering Division, King Abdullah University of Science and Technology (KAUST), Thuwal 23955­6900, Kingdom of Saudi Arabia

## Abstract

Flow and mass cytometry technologies can probe proteins as biological markers in thousands of individual cells simultaneously, providing unprecedented opportunities for reconstructing networks of protein interactions through machine learning algorithms. The network reconstruction (NR) problem has been well-studied by the machine learning community. However, the potentials of available methods remain largely unknown to the cytometry community, mainly due to their intrinsic complexity and the lack of comprehensive, powerful and easy-to-use NR software implementations specific for cytometry data. To bridge this gap, we present Single CEll NEtwork Reconstruction sYstem (SCENERY), a web server featuring several standard and advanced cytometry data analysis methods coupled with NR algorithms in a user-friendly, on-line environment. In SCENERY, users may upload their data and set their own study design. The server offers several data analysis options categorized into three classes of methods: data (pre)processing, statistical analysis and NR. The server also provides interactive visualization and download of results as ready-to-publish images or multimedia reports. Its core is modular and based on the widely-used and robust R platform allowing power users to extend its functionalities by submitting their own NR methods. SCENERY is available at scenery.csd.uoc.gr or http://mensxmachina.org/en/software/.

## INTRODUCTION

Every process in the cell, ranging from proliferation and differentiation to cell survival and death, results from a sequence of molecular interactions. These sequences participate in forming complex and interconnected interaction networks, known as *pathways*. High-throughput profiling technologies available to biologists today yield datasets suitable for studying biological pathways through statistical and mathematical modeling techniques (1). Flow cytometry, in particular, is a powerful technology that can assay proteins as biological markers in thousands of cells at single-cell resolution. Coupled with computational methods, the widespread use of flow cytometry has led to the significant improvement of our understanding of biological mechanisms, especially of the immune system (2). More recently, the introduction of mass cytometry significantly increased the number of parameters that can be assayed per cell, generating large and high-dimensional datasets (3). This advancement provides unprecedented opportunities for the study, identification and interpretation of molecular pathways through network reconstruction (NR) methods and any other modeling techniques that require high numbers of cell samples.

The problem of NR has been well-studied by the machine learning community and its use in biology is increasing, particularly for the identification of gene regulatory networks (4). In the cytometry field the first successful NR was realized over a decade ago (5). Since then, however, the potentials of these methods have been remaining largely unknown to the cytometry community, mainly due to their intrinsic complexity and the lack of user-friendly, powerful software implementations specific to cytometry data (6). The approach used by most biologists today for reconstructing pathways is typically reductionist: each interaction is tested in isolation and then mapped along with the other components of the network. NR methods may guide biologists in this task by suggesting a plausible, initial network of interactions, significantly reducing the demand on time and resources.

Here, we attempt to bridge the gap between the machine learning and single cell cytometry communities by presenting a web server called SCENERY (Single CEll NEtwork Reconstruction sYstem). SCENERY features standard cytometry data analysis methods coupled with NR algorithms in a user-friendly, on-line environment. As input, the server requires a cytometry dataset and a specification of the study design. Multimedia reports and ready-to-publish images are available as output. The core of the server is modular and based upon the widely-used and robust R platform. We emphasize that SCENERY has an open architecture that allows power users to extend its functionalities by submitting their own methods and become available to all its users. In this way, it can also be helpful to NR methodology researchers offering them a graphical user interface and an environment to develop and visualize their methods. Furthermore, technological advancements in flow- and mass-cytometry lead a continuously growing need for computational techniques that are able to extract new biological knowledge from high-throughput, high-dimensional cytometry data (2). Toward this direction, a variety of novel analysis approaches for data visualization and automatic population identification are becoming available (7). Our vision is to exploit SCENERY's modularity and R-based architecture for making it an umbrella service for all these different computational techniques in the near future.

To the best of our knowledge, the SCENERY web server is the first of its kind. Although basic cytometry data analyses are available in several other stand-alone software (8) or web services (9), none of them provides the user with advanced NR data analysis functionalities.

## SCENERY WEB SERVER

### Functionality

**Figure 1.**
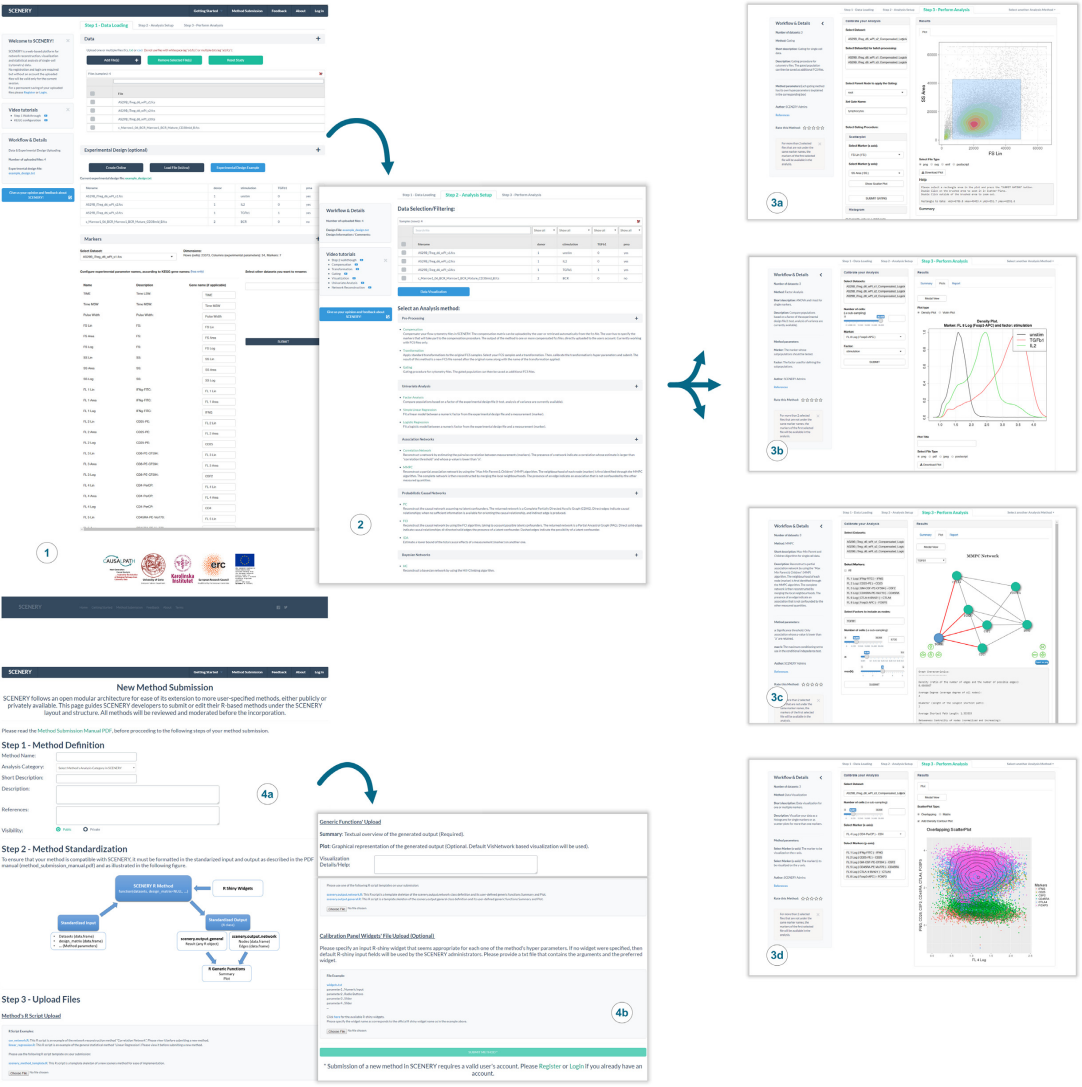
SCENERY workflow and functionalities. (1) At step 1, users submit data files and optional information about the experimental design. Access to the *Getting Started* section is always available from the top menu. (2) Step 2 allows overviewing the data and selecting an analysis to perform. SCENERY offers advanced machine learning methods on pre-processing, univariate analysis and NR. At step 3 users calibrate and perform the intended analysis i.e.: (3a) gate cell populations; (3b) compare between factor distributions; (3c) NR; (3d) data visualization; (4a and b) A notable functionality of SCENERY is its modularity. Following the web server standards power users can prepare and submit their own single-cell analysis methods. To guarantee the compatibility with the layout and structure of SCENERY, moderation of the submitted methods is performed offline by the server administrators.

SCENERY offers a wide range of data analysis methods including, (i) basic pre-processing methods, to allow users to transform, compensate and manually gate samples; (ii) univariate analysis methods such as regression and factor analysis and (iii) advanced machine learning methods for association and causal NR that identify interactions between the measured quantities. Performing an analysis in SCENERY is a straightforward process: at step 1, users upload their data and supply information regarding the experimental design; step 2 allows overviewing the data and selecting the analysis to perform. At step 3, the users define the markers to include in the analysis, eventually set the hyperparameters (all values that a user should be specifying before running a computational analysis) of the selected method and launch the execution (Figure 1). This pipeline is not restrictive in that the user can re-iterate across any step and change its settings; particularly, at step 3 users can reset the hyperparameters or run different methods until the intended analysis is complete. The results are presented to the user upon completion; alternatively, a web link to pending results is automatically created at runtime to let users access them at their convenience. A notable functionality of the web server is that the inferred NR results can be automatically contrasted against the biological database resource KEGG (Kyoto Encyclopedia of Genes and Genomes) (10): upon selection of a retrieved interaction by the user, all biological pathways containing one or more paths between the participating nodes (markers) are presented. In this way, users can evaluate the resultant associations or causal relations on the reconstructed networks. Results can be downloaded in various output formats. To help users familiarize with SCENERY, a *Getting Started* section is available that includes an *Interactive Tutorial, Introductory Material* and *Example Files* as sample data to load automatically and experience the offered functionalities. Though not mandatory, SCENERY features a password-protected login system and personal dashboard where registered users can permanently store data for future analyses.

### Data input

The web server's minimum input requirement is loading the file(s) containing the flow-/mass-cytometry data. These files can be either comma-/tab-separated text files or flow cytometry standard (FCS) files, which is a binary file format universally adopted by all cytometry software and hardware vendors. In this way SCENERY is able to accept existing cytometry data as produced and stored by most available third-party applications.

The uploaded data files may refer to different experimental conditions, distinguished by one or more experimental factors. The set of these factors constitute the study design that contains essential information used by SCENERY's NR tools. Experimental factor declaration in SCENERY can be made online or by uploading a text file according to the available template. Any number of qualitative (e.g. patient ID, cell type) or quantitative (e.g. activation/drug dosage) factors is supported, allowing SCENERY to accommodate all possible study designs.

Finally, the user can assign to each marker its corresponding gene name. These gene names will be subsequently used for contrasting NR results against online databases of molecular interactions.

### Featured analyses

#### Pre-processing

Correctly preparing the cytometry data is a fundamental, non-trivial step for meaningful analyses. SCENERY offers a set of pre-processing functionalities specific for cytometry data. *Compensation* for spectrally overlapping fluorescence signals acquired by flow cytometry can be attained by using the compensation matrix embedded in FCS files (acquisition-defined matrix) or any other matrix manually specified by the user. Several data *transformation* options are also available for improving *visualization* and cell population *gating* across the range of data. Accordingly, users can *gate* their data by using a practical and intuitive graphical interface, much alike other cytometry applications used routinely by many cytometry experts. Particularly, the user can visualize cell (sub-) populations on 2D scatterplots and hierarchically gate cell populations of interest by highlighting the corresponding area of the plot. The selected events can then be saved in separate FCS files ready to be analyzed further.

#### Univariate analysis

Associations between experimental factors and the distribution of a single marker can be assessed via *univariate* statistical tests. For example, differences in protein phosphorylation across different samples or cell populations can be assessed by *t*-test (2 samples) or ANOVA (multiple samples). In a similar way, the rate of change over time (i.e. a continuous variable) of a cellular/cytoplasmic protein can be quantified through *linear* or *logistic regression*.

#### Network reconstruction

NR methods are SCENERY's most distinctive feature. The rationale behind all these methods is unravelling the multivariate and complex relationships underlying single-cell cytometry data; these relationships (a.k.a. interactions) are represented as edges in networks where nodes stand for the measured quantities (usually proteins). The *semantics* of the interactions represented by the edges depends on the class of the NR method used. *Association networks* simply compute associations (e.g. correlations) between the measured quantities and retain as edges the associations exceeding a user-specified threshold. *Conditional association networks* compute associations between quantities conditioned on (a subset of) all other quantities. The resulting edges are supposed to be not confounded by any other measured quantity. *Causal networks* attempt to discover causal relationships between measured quantities (X, Y) in the form ‘X causes Y’, going beyond the classical associative paradigm. Some causal NR methods, e.g. the PC (named after its inventors Peter Spirted and Clark Glymour) algorithm (11), assume that no latent confounders exist and produce networks known as complete Complete Partially Directed Acyclic Graphs (CPDAGs). In these networks direct edges indicate direct causal effects, while indirect edges represent those that lack sufficient information for providing an orientation. Other methods, like the Fast Causal Inference (FCI) algorithm (12), are able to take into account the possibility of latent confounders and produce graphs known as Partial Ancestral Graphs (PAGs), where edges can indicate either direct causal relationships, presence of confounding factors or uncertainty in the orientation. For more details on the field of causal discovery, interested readers are referred to (6).

Notably, users may select any factor(s) from the study design to be included in the network, so that their influence can also be taken into account alongside between-marker interactions. Finally, all NR methods included in the web server have strong theoretical background, underwent extensive evaluations and were published in high-profile journals. Their use by the cytometry community through SCENERY and the collective experience which will be gathered on their performance can be a basis for refinement and adjustments on the peculiarities of cytometry data.

### Performing an analysis and output

When performing an analysis two panels are available to the user, a *calibration* panel and *results* panel. On the former the user may tune the hyperparameters and define the protein markers upon which the analysis should be performed. The latter is a tabular panel where, depending on the selected analysis, the respective method results are available in three different formats namely, textual *summary*; scatter, violin, density or interactive network visualization *plot* and/or multimedia *report*. Below every reconstructed network the basic graph characteristics are also reported using standard metrics and algorithms from graph theory (e.g. the graph's density, diameter, in- and out-degree, etc.). All plots are available for download either as publication-quality images (PNG, PDF, JPG or postscript) or standard graph-representation format (i.e. Graph Exchange XML Format, GEXF) that can be readily imported in Cytoscape for further processing (13). A multimedia report is also downloadable as an HTML file and includes the analysis details such as the input dataset, selected markers and hyperparameter settings, the textual summary of the method and the interactive network visualization.

### An extendable software architecture

SCENERY is a platform-independent web application built on R and PHP. Its interface is based on modern web development technologies (e.g. HTML5, CSS3 and Bootstrap Framework). Particularly, the R-Shiny web framework is used for wrapping each analysis method into an independent web application and to allow R functions to communicate across the stacks of the web server (see Supplementary Table S1 for a complete list of respective R functions).

One of the most distinct features that SCENERY offers, besides NR, is its open architecture by which it allows single-cell analysis method developers to extend its functionalities. A special HTML form found on the top menu is dedicated for such power users to complete and submit their own R implementations (Figure 1). Successful submission requires users to comply their method's input and output signatures with the web server standards which ensure the smooth integration to its structure. To this end a stepwise tutorial with comprehensive examples, illustrations, templates and downloadable instructions is available.

## USE-CASES

To showcase the offered web service we apply SCENERY on both a flow cytometry and mass cytometry dataset respectively. Our goal in both analyses is leveraging information on pathway mechanisms that these data may encompass. The input files from both analyses are provided as examples in the *Getting Started* section described above.

**Figure 2.**
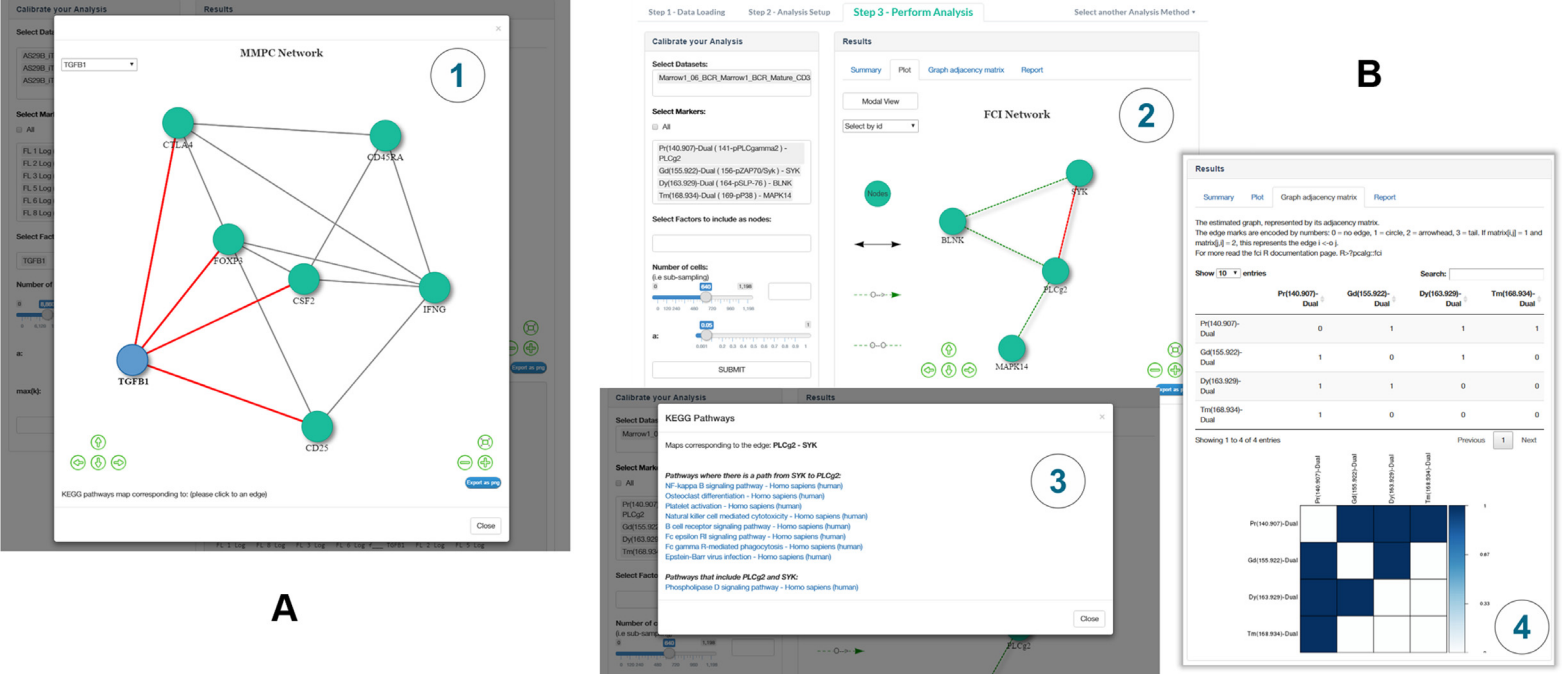
Example use-cases. (**A**) iTreg cells and control T cells were cultured and pre-gated on live CD4+ T cells as described in Supplementary Figure S1, using a subset of three samples (s1, unstimulated; s2, control stimulation + IL-2; s3, iTreg stimulation + IL-2 + TGF-β). The given markers were included in the analysis and the protein network as reconstructed by the MMPC algorithm is depicted. (**B**) Network reconstruction results on the B cell antigen-receptor (BCR) signaling data after using the Fast Causal Inference (FCI) causal NR algorithm. There are several options to explore NR results in SCENERY. (3) By clicking on any reconstructed edge the user is informed with active links about all molecular pathways in the KEGG database that include the respective nodes. Here, the maps that correspond to the edge between PLCγ2 and SYK are indicated. (4) Graphs are also displayed in matricial form for the user's convenience.

The flow cytometry data are derived from a study on human induced regulatory T cell (iTreg) generation. Here, naïve CD4+ T cells were either activated in the presence of IL-2 alone (control stimulation; sample ‘s2’), IL-2 + TGF-β (iTreg; ‘s3’) or left unstimulated (‘s1’) as described previously (14). On these three samples, we first employed SCENERY's pre-processing functionality to compensate for spillover according to the acquisition-defined matrix embedded in the .fcs files. Subsequently, the data were logicle transformed and gated on live CD4+ cells by a standard immunology gating strategy (see Supplementary Figure S1a and c). The gated data were submitted to the MMPC (Maximum Minimum Parents and Children) algorithm for NR analysis. Figure 2A shows the *Modal view* of the reconstructed network. The resulting edges indicate correlation between the respective proteins in a sense that both nodes have been selected in the parent–children set of each other. The reconstructed network indicates several established and recently identified associations of TGF-β leading to the upregulation of CD25, CTLA-4 and FOXP3 expression (Figure 2A and Supplementary Figure S1b and d)–well-known markers all upregulated in Tregs and positively associated to each other (14). At the same time, the inhibitory effect that TGF-β has under these conditions on the expression of the effector T cell cytokine GM-CSF (15) was also represented in the network (Figure 2A and Supplementary Figure S1b and d). The general effect of T cell activation that leads to upregulation of CD25, CTLA-4 and FOXP3 and down-regulation of CD45RA was also apparent through inclusion of the unstimulated sample along with the stimulated ones (Supplementary Figure S1b and d).

The mass cytometry data are publicly available from a study where 31 protein markers related to the human hematopoietic system were measured after stimulating bone marrow cells from two healthy donors with several activators to uncover distinct signaling mechanisms (16). These markers included intracellular phospho-proteins, the quantity of which directly relates to the activation state of the respective signaling proteins in the pathways studied. For this second use-case, we selected samples treated with B cell antigen-receptor (BCR) stimulus which is known to trigger a network of signaling cascades leading to several distinct outcomes. No pre-processing is required since the available public data are already pre-processed. The upper panel of Figure 2B illustrates the reconstructed network using the FCI causal NR method. The NR results capture a small part of the BCR signaling cascade highlighting well-known associations between phospho-SYK, phospho-BLNK, phospho-PLCγ2 and phospho-p38 proteins (17,18). As indicated in the lower panel of Figure 2B, SCENERY assists users in evaluating the reconstructed edges of the graph by offering a direct link to all pathways in the well-annotated KEGG database (10) where the respective nodes/protein markers are indicated as directly or indirectly associated.

## CONCLUSION

In this work, we present SCENERY, a web server specifically devised to allow researchers to apply standard pre-processing, statistical analysis, advanced visualization methods and NR methods on single-cell cytometry data. A part of this work was first introduced in (18). Since then, SCENERY has been developed into a complete flow-/mass-cytometry data analysis online toolkit. Especially, the fact that power users are welcome to extend its features by submitting their own single-cell analysis methods paves the way for SCENERY to become an open platform for single-cell data analysis.

In order to ensure a complete, efficient and robust platform for single-cell analysis, this work focused on the development of the modular architecture and the appropriate functionality after deriving feedback from experts in various relevant fields such as human—computer interaction, computational biology and particularly, cytometry. Moreover, one of our main goals is to render this type of analysis accessible, especially, to non-expert users in data analysis. This ensures an ease of selecting the appropriate pipeline and rapidly applying state-of-the-art computational methods and standard work-flows in single-cell analysis by avoiding the common and typically demanding programming and algorithmic overhead associated with such types of analyses. In the near future, SCENERY will be extended by adding other single-cell analysis tasks, such as clustering, dimensionality reduction and other common cytometry types of analyses by incorporating several popular computational techniques available in R such as Citrus, t-SNE and ACCENSE and also connection to other databases for network validation such as STRING (19–22), that could transform it into a multipurpose single-cell data analysis platform to the cytometry community.

## Supplementary Material

Supplementary Data
