# Supplementary material for: SCENERY: a web application for (causal) network reconstruction from cytometry data
Source: Nucleic Acids Res. 2017 May 19;45(Web Server issue):W270–5. doi: 10.1093/nar/gkx448 (PMC5570263; doi:10.1093/nar/gkx448)
Supplement: Supplementary Data [file gkx448_Supp.docx]

SUPPLEMENTARY TABLE 1

R methods employed for SCENERY’s analysis functionalities

| **Method** | **Analysis Category** | **Short Description** | **Result** | **R package/Reference** |
| --- | --- | --- | --- | --- |
| Data Visualization | Visualization | Visualization of single-cell measurements | Histograms, scatterplots, density-contour plots | *ggplot* <https://cran.r-project.org/web/packages/ggplot2>  *corrplot*  <https://cran.r-project.org/web/packages/corrplot>  *reshape2* <https://cran.r-project.org/web/packages/reshape2> |
| Transformation | Pre-processing | Transformation procedure for cytometry files | New FCS files | *flowStats*  <https://www.bioconductor.org/packages/release/bioc/html/flowStats.html> |
| Compensation | Pre-processing | Compensation procedure for flow cytometry files | New FCS files | *flowStats*  <https://www.bioconductor.org/packages/release/bioc/html/flowStats.html>  *flowCore*  <http://bioconductor.org/packages/release/bioc/html/flowCore.html> |
| Gating | Pre-processing | Gating procedure for flow cytometry files | R Shiny interactive plots, new FCS files | *flowStats*  <https://www.bioconductor.org/packages/release/bioc/html/flowStats.html>  *flowWorkspace* <http://www.bioconductor.org/packages/release/bioc/html/flowWorkspace.html> |
| Factor Analysis | Univariate statistical analysis | Population comparison based on experimental factors (t-test-anova) | Summary statistics, density plots, violin plots | *base R package* |
| Linear Regression | Univariate statistical analysis | Fits a linear model between a numeric experimental design factor and a measurement | Summary statistics, scatterplots with fitted regression lines | *base R package* |
| Logistic Regression | Univariate statistical analysis | Fits a logistic model between a numeric experimental design factor and a measurement | Summary statistics, scatterplots with fitted regression lines | *nnet*  <https://cran.r-project.org/web/packages/nnet> |
| Correlation | NR | Reconstructs an association network | Undirected graphs | *base R package*  *visNetwork*  <https://cran.r-project.org/web/packages/visNetwork/> |
| MMPC | NR | Reconstructs a conditional association network | Undirected graphs | *MxM* <https://cran.r-project.org/package=MXM>  *visNetwork*  <https://cran.r-project.org/web/packages/visNetwork/> |
| PC | NR | Reconstructs a causal network assuming no latent confounders | Partial directed graphs | pcalg <https://cran.r-project.org/web/packages/pcalg>  *visNetwork*  <https://cran.r-project.org/web/packages/visNetwork/> |
| FCI | NR | Reconstructs a causal network assuming latent confounders | Partial ancestral graphs | pcalg <https://cran.r-project.org/web/packages/pcalg>  *visNetwork*  <https://cran.r-project.org/web/packages/visNetwork/> |
| IDA | NR | Estimates possible total causal effects | Causal effects summary | *pcalg* <https://cran.r-project.org/web/packages/pcalg> |
| HC | NR | Reconstructs a Bayesian networks | Directed acyclic graphs | *bnlearn* <https://cran.r-project.org/web/packages/bnlearn/>  *visNetwork*  <https://cran.r-project.org/web/packages/visNetwork/> |


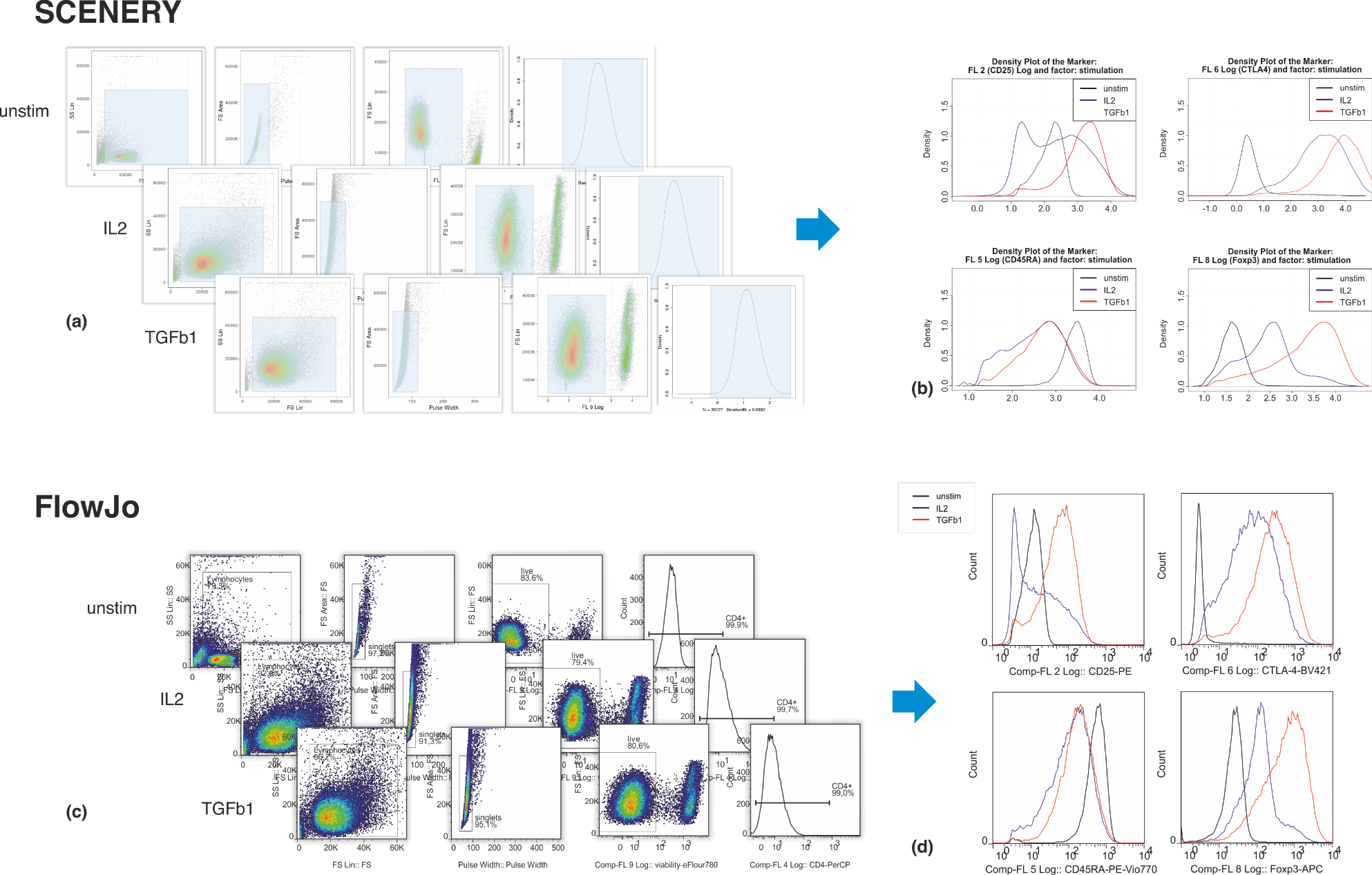


SUPPLEMENTARY FIGURE 1. Gating strategy for flow cytometry data from a study on human induced regulatory T cell (iTreg) differentiation. Naïve CD4+ T cells were activated for 6 days with T cell receptor stimulation (plate-bound anti-CD3 antibody) and co-stimulation (anti-CD28 antibody). For control stimulated cells, the cytokine IL-2 was added to these cultures (“IL2”), while for differentiation of iTregs, a combination of the cytokines IL-2 and TGF-β1 was added (“TGFb1”). As a further control sample, cells were left unstimulated (“unstim”). All samples were pulsed for 4 hours with Phorbol 12-myristate 13-acetate, Ionomycin and Brefeldin A to enable the detection of intracellular cytokines, and then stained and acquired on a flow cytometer as described (14). In brief, cells were first stained for surface CD4, CD25 and CD45RA expression, then with a fixable viability dye, and subsequently fixed, permeabilized and stained for FOXP3, GM-CSF, IFN-γ and CTLA-4. Samples were acquired on a CyAn ADP 9 Color Analyzer (Beckman Coulter) and compensation was performed automatically with the CyAn software (Summit) tool of the flow cytometer using single stained samples containing positive cells for the respective staining. (a-d) As an example, the gating strategy for the three samples, after applying the compensation matrix and logicle transformation, is shown. (a, c) First, lymphocytes were selected by fsc/ssc, then doublets were excluded. Subsequently, it was gated on live cells and then on CD4+ T cells.  (a) depicts the gating performed in SCENERY and (c) shows gating performed in the software FlowJo (Tree Star Inc.) for comparison. (b, d)  shows histograms of the given markers after pre-gating on live CD4+ T cells as shown in the left panel (a and c respectively). Unstimulated (black), control stimulated (blue) and iTreg (red) samples are shown as overlay (analyzed by SCENERY in (b) or FlowJo in (d) for the indicated markers.
